# Repercussion of Megakaryocyte-Specific Gata1 Loss on Megakaryopoiesis and the Hematopoietic Precursor Compartment

**DOI:** 10.1371/journal.pone.0154342

**Published:** 2016-05-06

**Authors:** Marjolein Meinders, Mark Hoogenboezem, Maaike R. Scheenstra, Iris M. De Cuyper, Petros Papadopoulos, Tamás Németh, Attila Mócsai, Timo K. van den Berg, Taco W. Kuijpers, Laura Gutiérrez

**Affiliations:** 1 Dept. of Blood Cell Research, Sanquin Research and Landsteiner Laboratory, Academic Medical Centre (AMC), University of Amsterdam (UvA), Amsterdam, the Netherlands; 2 Dept. of Molecular Cell Biology, Sanquin Research and Landsteiner Laboratory, AMC, UvA, Amsterdam, the Netherlands; 3 Dept. of Hematology, Hospital Clínico San Carlos, Instituto de Investigación Sanitaria San Carlos (IdISSC), Madrid, Spain; 4 Dept. of Physiology, Semmelweis University School of Medicine, Budapest, Hungary; 5 MTA-SE “Lendület” Inflammation Physiology Research Group of the Hungarian Academy of Sciences and Semmelweis University, Budapest, Hungary; 6 Emma Children’s Hospital, Academic Medical Centre (AMC), UvA, Amsterdam, the Netherlands; Emory University, UNITED STATES

## Abstract

During hematopoiesis, transcriptional programs are essential for the commitment and differentiation of progenitors into the different blood lineages. GATA1 is a transcription factor expressed in several hematopoietic lineages and essential for proper erythropoiesis and megakaryopoiesis. Megakaryocyte-specific genes, such as *GP1BA*, are known to be directly regulated by GATA1. Mutations in GATA1 can lead to dyserythropoietic anemia and *pseudo* gray-platelet syndrome. Selective loss of Gata1 expression in adult mice results in macrothrombocytopenia with platelet dysfunction, characterized by an excess of immature megakaryocytes. To specifically analyze the impact of Gata1 loss in mature committed megakaryocytes, we generated Gata1-Lox|Pf4-Cre mice (Gata1cKO^MK^). Consistent with previous findings, Gata1cKO^MK^ mice are macrothrombocytopenic with platelet dysfunction. Supporting this notion we demonstrate that Gata1 regulates directly the transcription of Syk, a tyrosine kinase that functions downstream of Clec2 and GPVI receptors in megakaryocytes and platelets. Furthermore, we show that Gata1cKO^MK^ mice display an additional aberrant megakaryocyte differentiation stage. Interestingly, these mice present a misbalance of the multipotent progenitor compartment and the erythroid lineage, which translates into compensatory stress erythropoiesis and splenomegaly. Despite the severe thrombocytopenia, Gata1cKO^MK^ mice display a mild reduction of TPO plasma levels, and Gata1cKO^MK^ megakaryocytes show a mild increase in Pf4 mRNA levels; such a misbalance might be behind the general hematopoietic defects observed, affecting locally normal TPO and Pf4 levels at hematopoietic stem cell niches.

## Introduction

GATA1 is a critical transcription factor for the differentiation of several hematopoietic lineages [1]. It is expressed in megakaryocytes [2], primitive and definitive erythroid cells [3], eosinophils [4], mast cells [5], dendritic cells [6] and Sertoli cells of the testis [7]. It belongs to the GATA family of zinc-finger transcription factors, which recognize the W(A/T)GATAR(A/G) DNA motif, and is located on the X-chromosome [8].

GATA1 regulates the transcription of lineage-specific genes contributing to differentiation, cell cycle regulation and survival of the hematopoietic lineages that express it [9–12]. In addition, it regulates the expression of target genes either by repressing or activating their transcription, depending on the transcription factor complex where it acts [13]. As an additional dimension of regulation, timely GATA1 levels during lineage commitment and differentiation are crucial for its proper action as master transcription regulator, and reciprocal regulation with the other hematopoietic GATA factors, such as GATA2 and GATA3, during lineage commitment and specification needs to be properly orchestrated [14–18].

In humans, several mutations affecting the GATA1 gene have been described, which lead to a broad spectrum of defects, including X-linked thrombocytopenia (XLT), X-linked thrombocytopenia with thalassemia (XLTT) and transient myeloproliferative disorder (TMD) [19, 20]. All disorders have in common mutations mostly located in the amino (N)-zinc finger region of GATA1, which might influence either DNA binding directly and/or the interaction with Friend of Gata1 (FOG1), leading to macrothrombocytopenia. Besides these deficiencies, patients with Down syndrome are more likely to develop an acquired, *i*.*e*. somatic, *GATA1* mutation leading to a truncated form of GATA1 (GATA1s). This short GATA1 isoform increases the possibility to develop acute megakaryoblastic leukemia (AMKL) [21].

Gata1 knockout mice die around day E10.5 of gestation due to severe anemia [22] and conditional ablation of Gata1 has shown its requirement in the erythro-megakaryocytic lineages in adult mice [23]. Mice with targeted mutations on the *Gata1* promoter affecting its expression, such as Gata-1.05/X female mice and ΔneoΔHS mice, display severe macrothrombocytopenia, an increased proliferation of megakaryocytes, and platelets with a defective response to collagen and vWF [2, 24]. From these studies, megakaryocytic Gata1 target genes were unveiled, such as *GP1BA*, *GP1BB* and *PF4* [25]. However, these mouse models are not tissue/lineage specific or display downregulation of Gata1 instead of complete loss.

The generation of tissue specific conditional mouse model tools, *i*.*e*. Pf4-Cre mice, makes possible the study of Gata1 loss specifically in the megakaryocytic lineage [26]. We have generated megakaryocyte-specific Gata1-Lox|Pf4-Cre mice in order to study not only the direct consequences of Gata1 depletion in committed megakaryocytes but its repercussion on the general hematopoietic compartment. Consistent with previous findings, late ablation of Gata1 in the megakaryocytic lineage resulted in macrothrombocytopenia and platelets that are functionally impaired. Some of the defective responses in platelets were due to a defect on receptor expression, *i*.*e*. integrin β1, vWF-R. However, other defects appeared to happen downstream other receptors, *i*.*e*. Clec2 and GPVI. We have identified Syk, a pivotal tyrosine kinase that functions downstream of Clec2 and GPVI receptors in megakaryocytes and platelets, as a *bona fide* Gata1 target. Interestingly, we describe an additional aberrant megakaryocyte differentiation stage in bone marrow and spleen. Furthermore, we show that these mice, and most likely due to the platelet production defect and concomitant stress megakaryopoiesis caused by Gata1 loss in the megakaryocytic lineage, display misbalance of their multipotent progenitor and erythroid compartments and present with compensatory extramedullary hematopoiesis with splenomegaly.

## Materials and Methods

### Mice

Gata1-lox mice [27] were crossed with Pf4-Cre mice [26] and maintained in the Dutch Cancer Center (NKI) animal facility under specific pathogen-free conditions and following the institutional ethical committee guidelines.

Syk-lox animals (*Syk*^tm1.2Tara^) [28], obtained from Professor Alexander Tarakhovsky (The Rockefeller University, New York, USA), were crossed to Pf4-Cre mice [26]. The genotyping was carried out by using allele-specific PCR. The efficacy and specificity of cell-specific Syk deletion was tested on cell lysates by Western blotting (data not shown). The experimental animals were kept in individually sterile ventilated cages (Tecniplast) under specific pathogen-free conditions until use, when they were transferred to the conventional facility. Animal experiments made on these cell-specific Syk-deleted mice were approved by the Animal Experimentation Review Board of the Semmelweis University.

Mice were anesthetized with isofluoran, bled by heart puncture, euthanized by cervical dislocation and dissected for bone marrow and spleen collection.

### Blood analysis and platelet functional assays

Blood was drawn by heart puncture and collected in heparin-coated vials (Sarstedt, Nϋmbrecht, Germany). Blood parameters were determined on a scil Vet abc Plus+. FCA was performed as described [29]. Platelet adhesion to collagen at physiological shear rate was performed as described [30]. In brief, whole blood was reconstituted to the same platelet concentration amongst genotypes, and was perfused over collagen-coated slides (Horm-Collagen type I, 100 μg/ml) at a shear rate of 1300^s-1^. After labeling platelets with CD61-FITC conjugated antibody, photos were taken at 200x magnification with an EVOS microscope. Coverage of fluorescence was quantified with ImageJ software.

### Flow cytometry

Platelets or bone marrow single cell suspensions were stained for flow cytometry analysis as previously described [30, 31]. Antibodies used were: CD61-FITC, CD41-PE, Sca1-PECy7, CD34-FITC, Lin-cocktail-APC, CD16/CD32-PE, cKit-PerCP, CD71-FITC, Ter119-V405 (BD Pharmingen, Oxford, United Kingdom), Clec2–FITC (AbD Serotec, Kidlington, United Kingdom), CD42a-FITC, CD42b-DL649, CD42c-FITC (Emfret, Wurzburg, Germany), GPVI-PE (R&D, Abingdon, United Kingdom), CD9-PE, CD31-PECy7 (Abcam, Cambridge United Kingdom) and CD49b-PB (BioLegend, San Diego, CA).

### Mouse plasma TPO ELISA

TPO levels were measured on the plasma of Gata1cKO^MK^ and WT^lox^ mice with a Quantikine Mouse Thrombopoietin ELISA Kit (R&D Systems), following the manufacturer´s guidelines.

### Bone marrow-derived megakaryocyte cultures

Megakaryocyte cultures were performed as previously described [30, 31].

### RNA

CD61^+^CD41^+^CD49b^+^ cells were sorted from megakaryocyte cultures on a FACS ARIA (BD Bioscience) and used for RNA extraction using Trizol (Ambion, Life Technologies). cDNA was prepared from 1μg RNA with Superscript III first strand (Invitrogen). Expression levels of mRNAs were analyzed by quantitative real-time PCR (qPCR) using SYBR green on an Applied Biosystems StepOne RT-qPCR system (Life Technologies). All reactions were performed in triplo.

Gene expression levels were calculated with the 2^-ΔCT^ method [32]. Target gene expression was normalized to Gapdh expression. Primers used were:

Gata1 5’-CAGTCCTTTCTTCTCTCCCAC-3’ and 5’-GCTCCACAGTTCACACACT-3’;

PU.1 5’-TCTTCACCTCGCCTGTCTT-3’ and 5’-TCCAGTTCTCGTCCAAGCA-3’;

Syk 5’-TCACAACAGGAAGGCACAC-3’ and 5’-GAGTGGTAATGGCAGAGGTC-3’;

Pf4 5’-CTGCGGTGTTTCGAGGCCTCC-3’ and 5’-accagcgctggtgacagcaa-3’;

Gapdh 5’-CCTGCCAAGTATGATGACAT-3’ and 5’-GTCCTCAGTGTAGCCCAAG-3’.

### Protein

Approximately 10^9^ platelets were lysed in 500 μl RIPA buffer (150 mM NaCl, 1% NP-40, 0.5% Deoxycolate, 0.1% SDS, 50 mM Tris HCL, pH 7.5). Proteins were separated by SDS-PAGE gel electrophoresis and transferred to PVDF membranes and incubated following standard procedures. Antibodies used were Anti-Gata1 (sc-265, Santa Cruz), Anti-Syk (sc-573, Santa Cruz), Anti-Gapdh (MAB374, Merck Millipore), and secondary antibodies IRDye 680 goat anti-mouse IgG and IRDye 800CW donkey anti-mouse IgG (926–32220 and 926–32212, respectively, LI-COR Biosciences). Western blot membranes were quantified using Odyssey LI-COR Imaging system.

### Chromatin Immunoprecipitation (ChIP)

Chromatin Immunoprecipitations were performed as previously described [33] using anti-Gata1 antibody (ab11963, Abcam) and protein A magnetic beads (10002D, Life Technologies). Enrichment of a DNA region of interest was measured by qPCR. Primers used were:

Syk GATA positive (+) amplicon:

5’-TCACAACAGGAAGGCACAC-3’ and 5’-GAGTGGTAATGGCAGAGGTC-3’;

Gp1ba GATA positive (+) amplicon:

5’-GCTGATAAGAGCCTTTGCC-3’ and 5’-GGGAGGAAATGACAACCTG-3;

Cd9 GATA negative (-) amplicon:

5’-ACCGTGCTCAACTAAGTGCG-3’ and 5’-AGATTCCCGAGGCAAGTCTG-3’.

### Statistical Analysis

We represent average and standard error of the mean (SEM) of at least three mice per genotype or experiments unless otherwise indicated. We applied two-tailed Student’s *t*-tests to calculate statistical significance.

## Results

**Fig 1:**
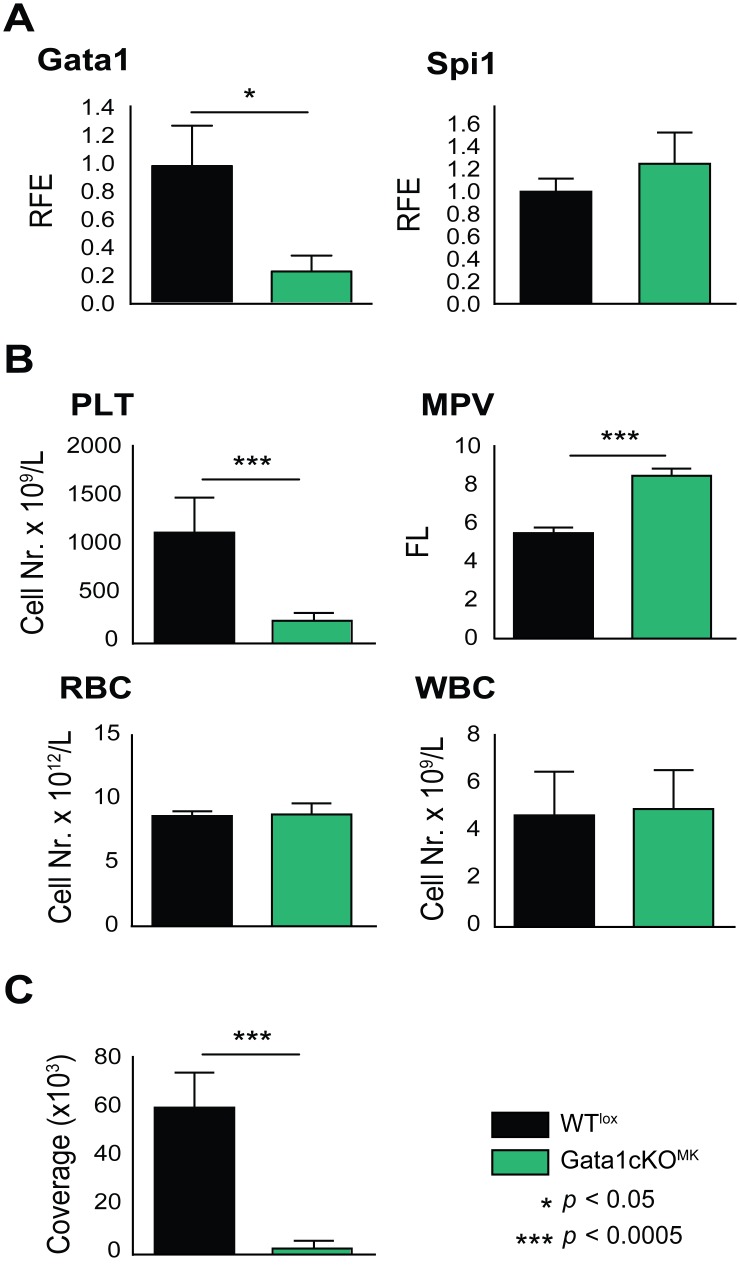
Gata1cKO^MK^ mice display severe macrothrombocytopenia and platelet dysfuncion while other blood cell counts are unaffected. **(a)** Gata1 and PU.1 (Spi1) mRNA expression levels in Gata1cKO^MK^ and WT^lox^ cultured megakaryocytes. PU.1 (Spi1) levels are normal in Gata1cKO^MK^ mice. RFE, relative fold enrichment. (b) Blood parameters of Gata1cKO^MK^ and WT^lox^ mice at 8–12 weeks of age. PLT, platelets; MPV, mean platelet volume; RBC, red blood cells; WBC, white blood cells. (c) Platelet adhesion to collagen under physiological shear rate. Whole blood from WT^lox^ and Gata1cKO^MK^ mice was perfused over collagen-coated slides. Adhered platelets were visualized with CD61-FITC conjugated antibody, and immunofluorescence coverage of slides was quantified, and is represented.

To investigate the role of Gata1 in late megakaryopoiesis, Gata1-lox|Pf4-Cre mice were generated [26, 27]. From now on we refer to these mice as Gata1cKO^MK^, and to control Gata1-lox littermates as WT^lox^. To quantify Gata1 reduction in Gata1cKO^MK^ mice, *Gata1* mRNA levels were quantified in cultured bone marrow megakaryocytes. On average, an 80% reduction of *Gata1* in megakaryocytes was observed, while the levels of transcription factor PU.1 (Spi1) were not affected (Fig 1a). Consistent with previous reports [2, 24, 25], Gata1cKO^MK^ mice suffer from severe macrothrombocytopenia, while the red and white blood cell numbers are unaffected (Fig 1b). Furthermore, platelets were impaired to adhere and to form thrombi over collagen-coated slides at physiological shear rate (Fig 1c).

### Malfunction of Gata1cKO^MK^ platelets

**Fig 2:**
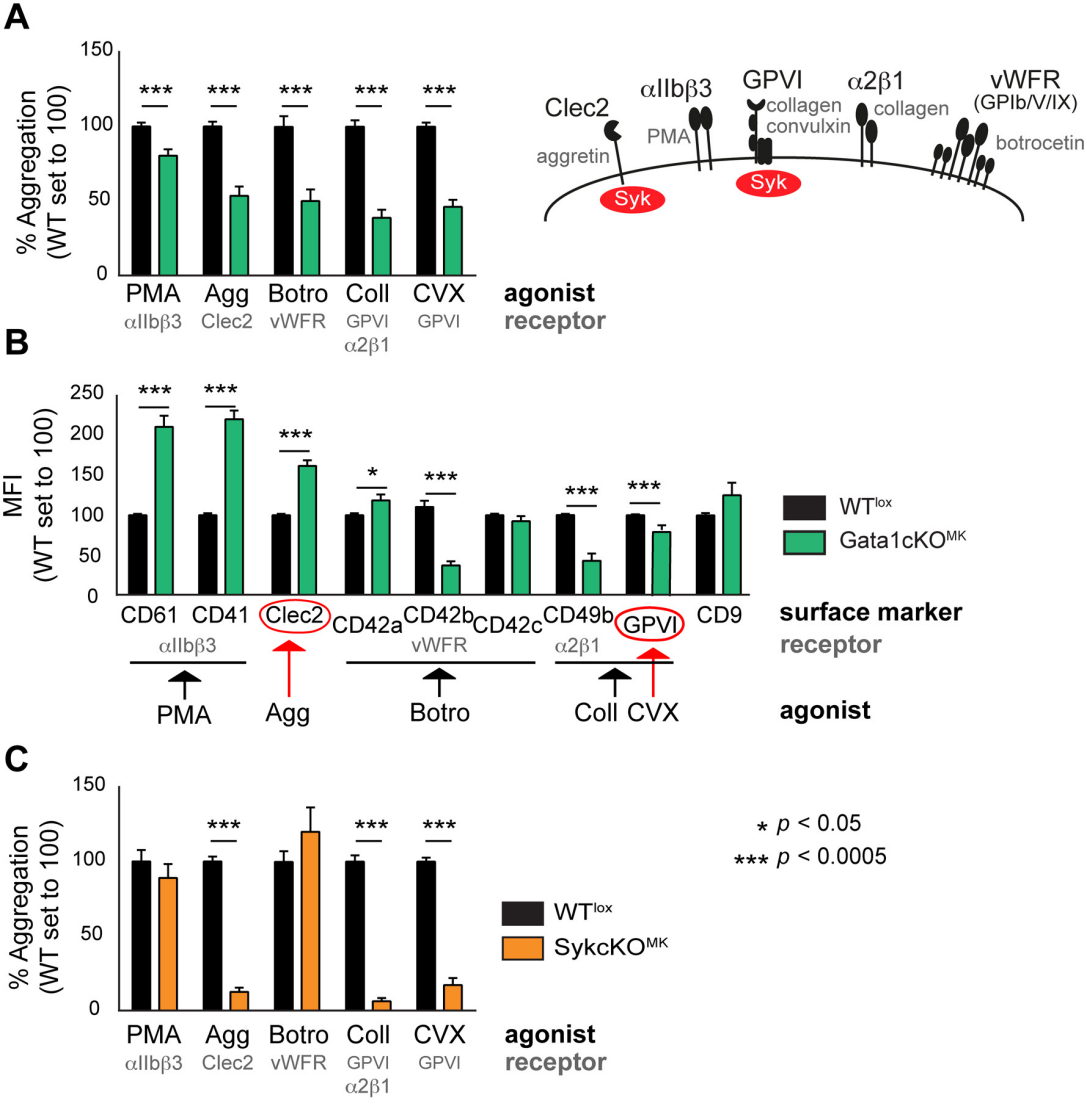
Functional analysis of platelets from Gata1cKO^MK^ and SykcKO^MK^ mice show overlapping defects. (a) Flow cytometry-based platelet aggregation assay (FCA) shows the aggregation capacity of platelets when stimulated with different agonists. Gata1cKO^MK^ and WT^lox^ platelets were studied. PMA, phorbol myristate acid; Agg, aggretin; Botro. Botrocetin; Coll, collagen; CVX, convulxin. (b) MFI of receptors expressed on Gata1cKO^MK^platelets, relative expression of a given receptor in WT^lox^ platelets was set to 100. For clarification: CD61 (Itgb3), CD41 (Itga2b), CD42a (GPIX), CD42b (Gp1ba), CD42c (Gp1bb), CD49b (Itga2). (c) Flow cytometry-based platelet aggregation assay (FCA) shows the aggregation capacity of SykcKO^MK^ and WT^lox^ platelets when stimulated with various agonists as described above.

It has been previously reported that platelets derived from ΔneoΔHS mice are dysfunctional [34], and consistently, we observed a dramatic defect of Gata1cKO^MK^ platelets using perfusion assay (Fig 1c). To increase knowledge on the platelet dysfunction caused by Gata1 loss in megakaryocytes in Gata1cKO^MK^ mice, we measured platelet aggregation using FCA, which allows the study of the contribution of single receptors to the aggregation process [29]. We observed that upon stimulation with aggretin (Clec2 agonist), collagen (GPVI and α2ß1 agonist), convulxin (GPVI agonist), and botrocetin (GPIb/V/IX agonist), platelet aggregation responses were severely reduced, whereas responses were only mildly affected when stimulated with PMA (PKC agonist that triggers αIIbß3 integrin-dependent aggregation; Fig 2a). The reduced aggregation capacity upon stimulation with botrocetin and collagen could be explained by the decreased expression of receptors/subunits, as previously reported (CD42b and CD49b; Fig 2b) [25, 35, 36]. In addition, the lower collagen response might also be due to a defective GPVI signaling response, as GPVI is described as the major collagen receptor [37]. Interestingly, the expression of Clec2 was not decreased, and GPVI expression was only minimally affected, which could therefore not explain the prominent reduction in aggregation when stimulated with aggretin, convulxin or collagen, respectively.

Since GPVI and Clec2 receptors share Syk-dependent signaling pathways [38], we next aimed at evaluating whether those responses are indeed Syk-dependent. In order to do that, we generated Syk-lox|Pf4-Cre mice. From now on we refer to these mice as SykcKO^MK^, and to control Syk-lox littermates as WT^lox^. We measured platelet aggregation using FCA as performed above, and observed that upon stimulation with aggretin (Clec2), collagen (GPVI and α2ß1, being GPVI the major collagen receptor) and convulxin (GPVI), platelet aggregation responses were almost absent, while the aggregation response was normal when stimulated with PMA (αIIbß3) and botrocetin (GPIb/V/IX; Fig 2c). This result corroborates the notion that Clec2 and GPVI share Syk-dependent signaling pathways [38], and confirms the overlap of Clec2- and GPVI-dependent responses with Gata1cKO^MK^ platelets, which prompted us to investigate whether Syk expression was affected in Gata1cKO^MK^ mice.

### Syk transcription is regulated by Gata1

**Fig 3:**
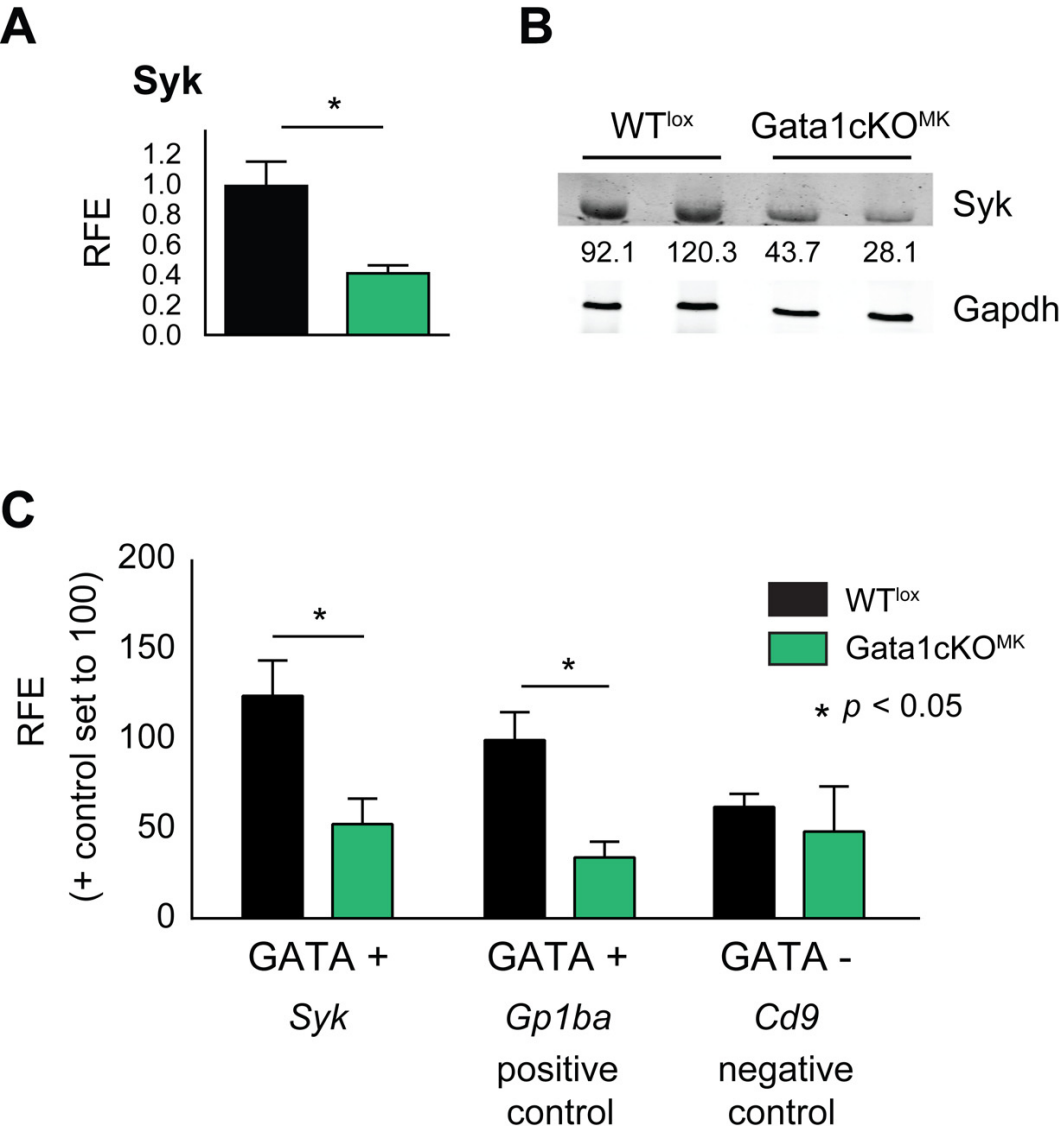
Gata1 regulates Syk expression. **(a)** Syk mRNA expression levels in Gata1cKO^MK^ and WT^lox^ cultured megakaryocytes measured by qRT-PCR. (b) Syk protein levels in Gata1cKO^MK^ and WT^lox^ platelets, analyzed by Western blotting. Syk expression level normalized to loading control Gapdh is indicated, setting the average expression levels of Syk in WT^lox^ platelets to 100. (c) Chromatin immunoprecipitation (ChIP) assay showsGata1 binding to the *Syk* promoter in WT^lox^ compared to Gata1cKO^MK^ (background control) cultured megakaryocytes. A GATA positive (+) site on the promoter of the known target *Gp1ba* (CD42b) was used as positive control and a GATA negative (−) site on the promoter of *Cd9* was used as negative control.

To investigate if Syk transcription is affected in the megakaryocytic lineage of Gata1cKO^MK^ mice, mRNA and protein expression levels were measured in cultured sorted Gata1cKO^MK^ megakaryocytes and platelets, respectively (Fig 3a and 3b). A significant reduction in both Syk mRNA and protein levels was observed, suggesting that Gata1 could regulate its expression. We identified a WGATAR binding site ≈ 600bp upstream of the *Syk* gene start codon and performed Gata1 ChIP experiments on WT^lox^ and Gata1cKO^MK^ megakaryocytes (as background control) to elucidate whether Gata1 interacts directly with the *Syk* promoter.

We took as positive control a GATA positive (+) amplicon (≈-300bp) at the *Gp1ba* promoter (known Gata1 target) and as a negative control a GATA negative (−) amplicon (≈-600bp) at the *Cd9* promoter. Gata1 ChIP results show enrichment at the GATA site in the *Syk* promoter in WT^lox^ megakaryocytes, similarly to the positive control, while it does not enrich above background at the GATA negative (−) site in the *Cd9* promoter (Fig 3c). Therefore, this data corroborates the notion that Syk is a *bona fide* Gata1 target.

### Aberrant megakaryocyte maturation in Gata1cKO^MK^ mice

**Fig 4:**
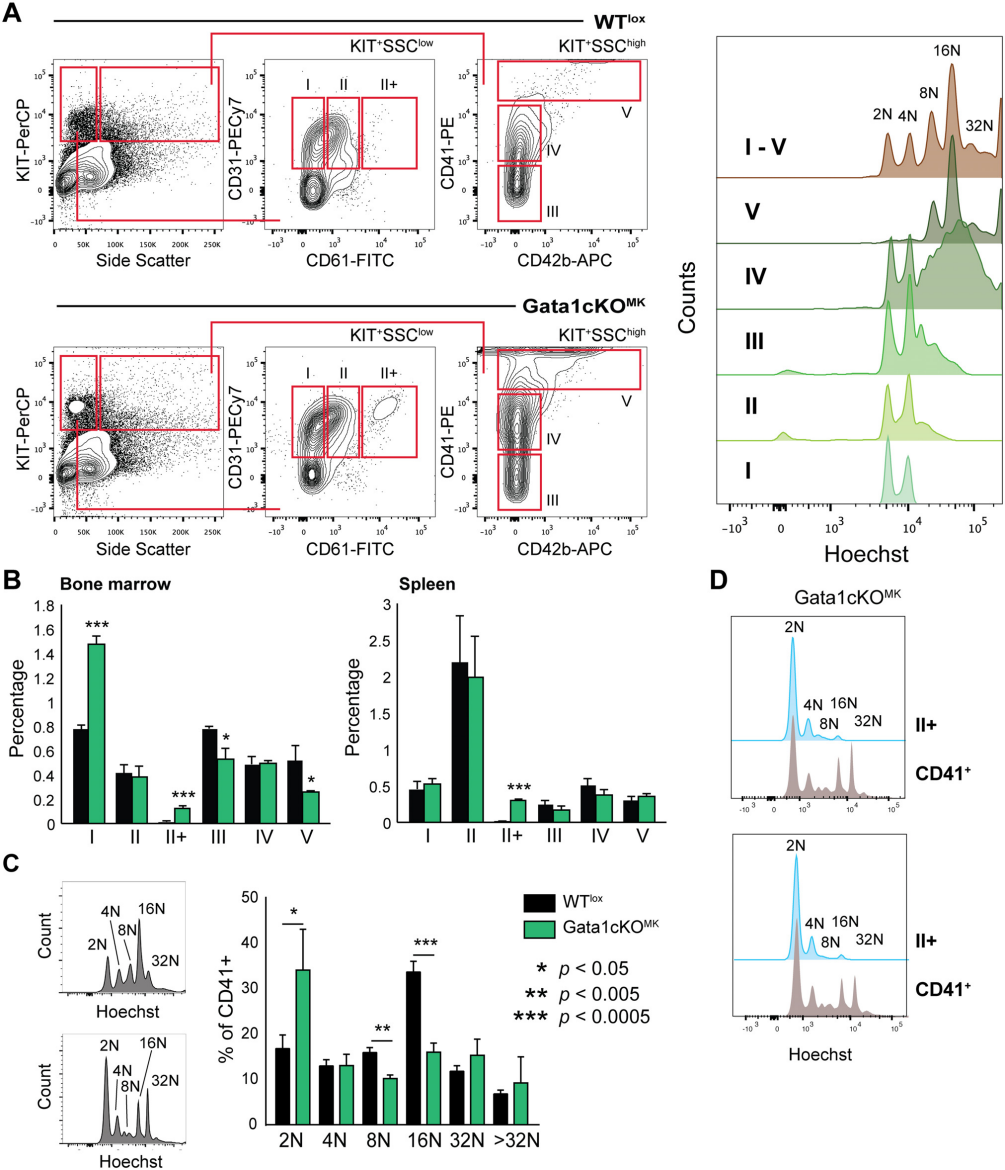
Gata1cKO^MK^mice have a defect in megakaryopoiesis. (a) Gating strategy to identify megakaryocytes at consecutive stages of differentiation in the bone marrow and the spleen based on surface marker expression CD31, CD61, CD41 and CD42b. The dot plot depicts the extra population, named II+ found exclusively in Gata1cKO^MK^ bone marrow. On the right we show the ploidy status of the individual subpopulations, thereby justifying our gating strategy. (b) Percentage of megakaryocytes at consecutive stages of differentiation (I-V) of nucleated bone marrow and spleen cells. (c) Ploidy staining of CD41-positive bone marrow and spleen megakaryocytes. The right bar graph depicts ploidy staining of CD41+ in bone marrow. (d) Ploidy status of gated II+ megakaryocyte differentiation stage vs total CD41+ cells in two representative Gata1cKO^MK^ bone marrow samples.

Megakaryocyte maturation was determined based on receptor expression profile in the bone marrow and the spleen. Five consecutive stages (I-V) of megakaryocyte maturation could be identified, as previously described, whereby subtype I contains the most immature and V the most mature megakaryocytes (for gating strategy see Fig 4a) [31].

To validate the differentiation status of our megakaryocyte subpopulations based on flow cytometry, the ploidy status of the individual subpopulations was analyzed. We observed a positive correlation of ploidy level with maturation progression following stages I to V, which in our view validates this megakaryocyte differentiation gating strategy [31].

Compared to WT^lox^ littermates, and as previously reported, we identified a shift towards more immature megakaryocytes in the bone marrow, *i*.*e*. a relative increase in population I and a decrease in populations IV-V (Fig 4b). Interestingly, we identified an additional subpopulation exclusively in the Gata1cKO^MK^ samples, indicated as II+, in which megakaryocytes with low SSC characteristics express higher levels of CD61. In concordance with this, and similarly to what has been described in other Gata1 deficient mouse models, when analyzing the ploidy status of CD41+ megakaryocytes in the bone marrow, we observed that there was an increase of 2N megakaryocytes, and the normal 16N profile was lost (Fig 4c). Furthermore, ploidy analysis of the II+ population revealed that this population was composed of mainly of megakaryocytes at 2N and 4N ploidy level, with a low percentage of 8N-16N, similarly to what we observe in population II in WT^lox^ samples, supporting the notion that the II+ population identified in the Gata1cKO^MK^ samples are megakaryocytes at stage II that start expressing higher (timely inappropriate) levels of CD61 (Fig 4d).

In contrast to human, the mouse spleen is known to be a secondary hematopoietic organ for megakaryopoiesis and erythropoiesis, whereby the output of erythroid cells and platelets increases during stress hematopoiesis [39]. Although we did not notice a change in any of the megakaryocyte progenitor subpopulations in the spleen, we did identify the II+ subpopulation, comparable to the bone marrow (Fig 4b). However, we cannot conclude whether this aberrant megakaryocytic stage is dependent on Gata1 loss, or a consequence of the thrombocytopenia or stress megakaryopoiesis itself.

### Misbalance of multipotent progenitors in the bone marrow of Gata1cKO^MK^ mice

**Fig 5:**
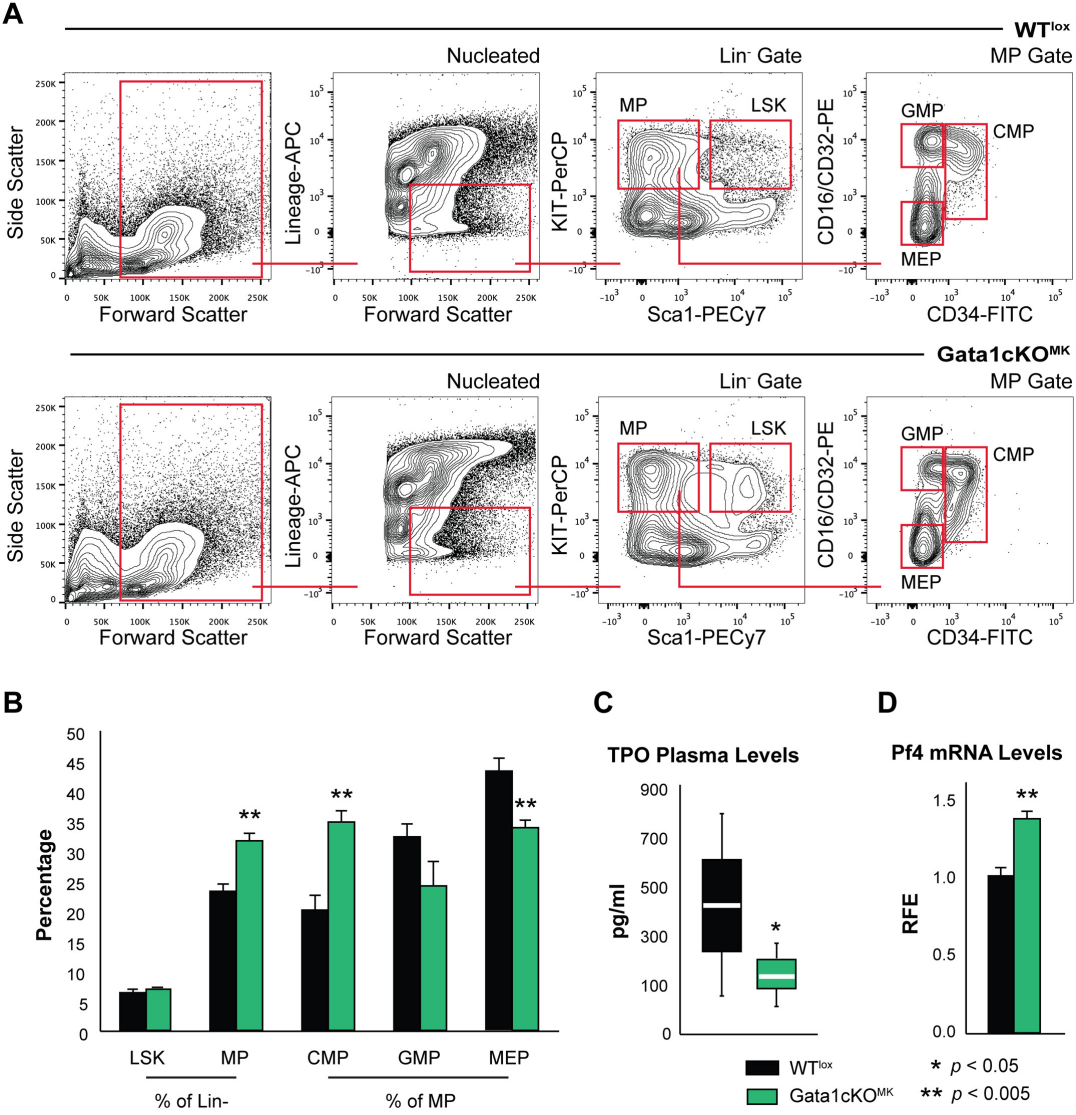
Gata1cKO^MK^ mice have a defect in the hematopoietic early precursor compartment (a) Flow cytometry analysis of the stem cell and committed progenitor compartment. LSK, Lin^-^|Sca-1^+^|Kit^+^ cells; MP (Lin^-^|Sca-1^-^|Kit^+^), multipotent progenitors; CMP (MP gate—CD34^+^|CD16/CD32^mid^), common myeloid progenitor; GMP (MP gate—CD34^-^|CD16/CD32^+^), granulocyte-monocyte progenitor; MEP (MP gate—CD34^-^|CD16/CD32^-^), megakaryocyte-erythroid progenitor. (b) Percentage of the different hematopoietic progenitors. Absolute cell number of bone marrow megakaryocytes at consecutive stages of differentiation [31]. The dot plot depicts the extra population, named II+ found exclusively in Gata1cKO^MK^ bone marrow. (c) Whisker/Box plot depicts plasma TPO levels from Gata1cKO^MK^ and WT^lox^ blood samples, as measured by ELISA. At least 5 mice were analyzed per genotype. (d) qPCR analysis of Pf4 mRNA expression levels in cultured bone marrow derived Gata1cKO^MK^ and WT^lox^ megakaryocytes.

To investigate the repercussion of Gata1 deletion in megakaryocytes on the hematopoietic compartment, we analyzed the distribution of hematopoietic progenitors in the bone marrow based on receptor surface expression whereby nucleated, Lin^-^|Sca-1^+^|Kit^+^ cells were identified as LSK and Lin^-^|Kit^+^|Sca-1^-^ as multipotent progenitors (MP). A further subdivision of MPs was made based on CD16/CD32 and CD34 expression, whereby we could differentiate between the common myeloid progenitor (CMPs), granulocyte-macrophage progenitor (GMP) and megakaryocyte-erythroid progenitor (MEP) (see Fig 5a for gating strategy).

This revealed that Gata1cKO^MK^ mice display a misbalance in hematopoietic progenitors, *i*.*e*. an increase in the total number of myeloid progenitors (MP). This increase is mainly due to an increase in CMP cells at the expense of GMP and MEP cells (Fig 5b). This could be an indirect effect of the chronic thrombocytopenia displayed by Gata1cKO^MK^ mice, however, we could not exclude other possibilities implicit to the genetic strategy as to affect the balance of hematopoietic progenitors in our system. Severe thrombocytopenia is accompanied by a raise in plasma thrombopoietin (TPO) levels, when the megakaryocyte-platelet mass is reduced, and it is known that excess TPO has a direct effect on hematopoietic stem cells [40]. Contrary to what could be anticipated, TPO plasma levels were mildly reduced in Gata1cKO^MK^ as compared to WT^lox^ mice (Fig 5c). This suggests that despite the severe thrombocytopenia, the megakaryocyte-platelet mass is increased, due to the overgrowth of immature megakaryocyte populations identified in the bone marrow in Gata1cKO^MK^ as compared to WT^lox^ mice (Fig 4b). On the other hand, it has been reported that Pf4 produced by megakaryocytes in the bone marrow niche affects hematopoietic stem cell quiescence and therefore performance [41], and since Pf4 is a Gata1 target gene [25, 42], we aimed at analyzing Pf4 mRNA levels in bone marrow derived Gata1cKO^MK^ megakaryocytes. As shown in Fig 5d, Pf4 was significantly, although mildly, upregulated in Gata1cKO^MK^ cultured megakaryocytes. Therefore, we can conclude that Pf4 production is not deficient in these megakaryocytes.

### Twinned compensatory stress erythropoiesis that accompanies the stress megakaryopoiesis of Gata1cKO^MK^ mice

As show in Fig 5, we identified alterations in the hematopoietic compartment, where we noticed and increase in CMP and a decrease in GMPs and MEPs. Although the red blood cell count in Gata1cKO^MK^ mice was normal (Fig 1b), we were interested on the repercussion of the defective platelet production on erythroid differentiation.

**Fig 6:**
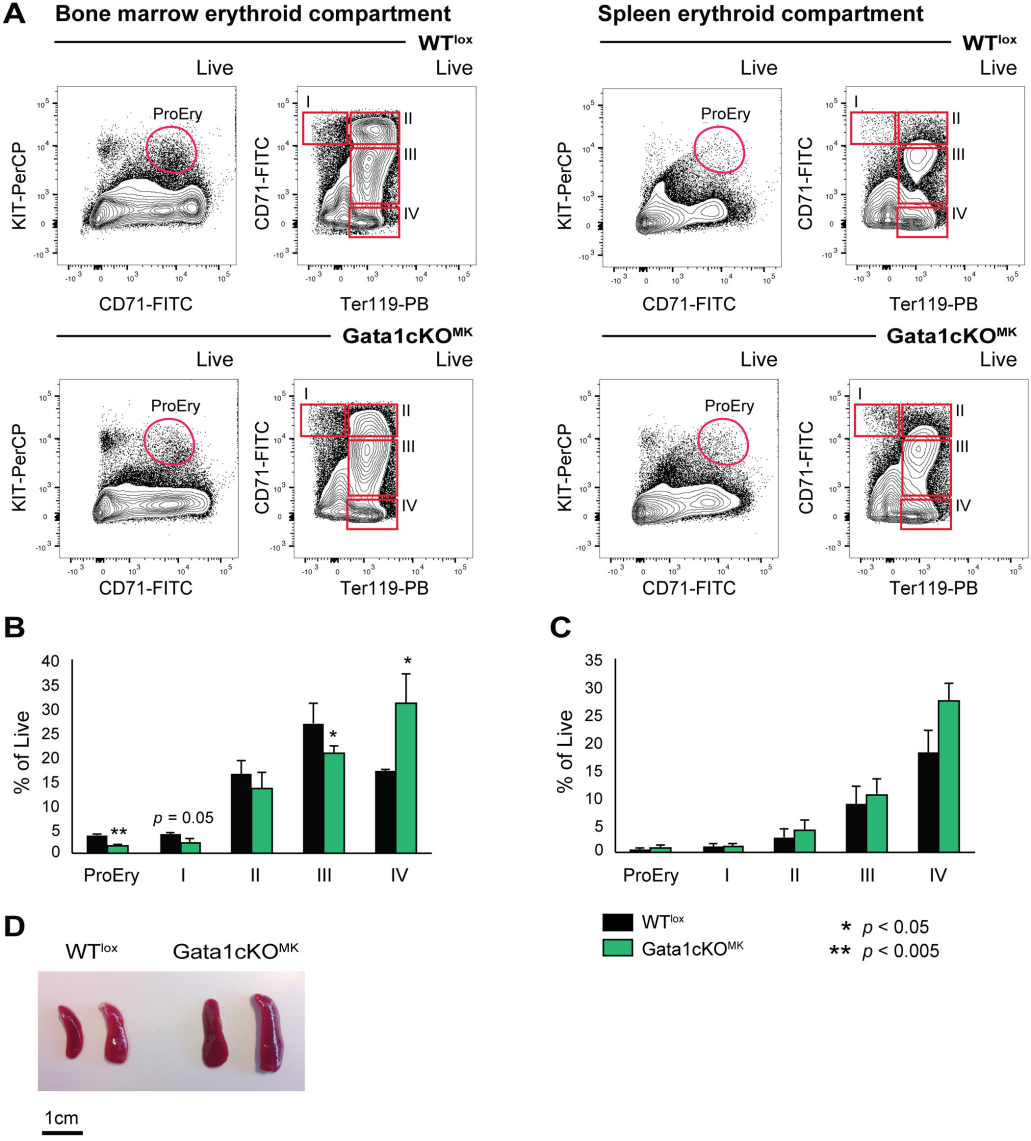
Gata1cKO^MK^mice show alterations in the erythroid compartment. (a) Gating strategy to identify erythrocytes at consecutive stages of differentiation in the bone marrow and the spleen based on surface marker expression KIT, CD71 and Ter119. (b) Percentage of reticulocytes at consecutive stages of differentiation of live cells. Left graph depicts the bone marrow compartment, right the splenic compartment. (c) Photograph of representative spleens from Gata1cKO^MK^ and control mice shows the splenomegaly that Gata1cKO^MK^develop.

Based on surface expression of CD71 and Ter119, as described by Socolovsky *et al* [43], four consecutive erythroid differentiation stages can be identified, whereby I is the most immature erythroid progenitor and IV the most mature erythroid progenitor; addition of KIT to the panel allows the detection of proerythroblasts, which are KIT^+^ CD71^+^ (for gating strategy see Fig 6a). The bone marrow erythroid compartment showed a clear shift towards more mature erythroid cells, *i*.*e*. in population IV and a decrease in the more immature erythroid populations, *i*.*e*. I-III (Fig 6b). The splenic erythroid compartment did not show overt changes in the distribution of I-IV stages, although there was a tendency to an increase in mature erythroid cells (stage IV, Fig 6c). However, and although the RBC counts were normal, these mice present with splenomegaly. This suggests that there is compensatory extramedullary hematopoiesis/erythropoiesis, probably due to the indirect exhaustion in MEPs caused by the described thrombocytopenia that suffer these mice (Fig 6d). Therefore, incapacitated megakaryopoiesis on a chronic manner (stress megakaryopoiesis) due to Gata1 loss in mature megakaryocytes, influences the early hematopoietic and erythroid compartments inducing compensatory extramedullary hematopoiesis/erythropoiesis.

## Discussion

Gata1 is a key transcription factor involved in both erythroid and megakaryocyte development and essential for terminal differentiation of these lineages. Since Gata1 knockout mice are lethal due to severe anemia [22], different *Gata1* targeted mouse models were generated to investigate the role of Gata1 in adult mice, including ΔneoΔHS [2] and Gata1.05 mice [24]. Although these models were fundamental in identifying the critical role of Gata1 in megakaryopoiesis, they are not restricted to the megakaryocytic lineage or they pose a model for the downregulation of the expression levels of Gata1 without gene ablation. Therefore, we generated Gata1-lox|Pf4-Cre mice, since Pf4-Cre are a generally accepted Cre mouse model to induce megakaryocyte/platelet specific gene recombination [26], to investigate the function of Gata1 in mature megakaryocytes. Previous reports have shown that this system could have some leakage in a minor population of circulating leukocytes, a subset of monocytes and macrophages and in epithelial cells of the colon; however, Gata1 is scarcely expressed in these cells, thus we exclude major effects from recombination of Gata1 in these lineages [44, 45].

Comparable to the ΔneoΔHS and Gata1.05 mouse models, Gata1cKO^MK^ mice show severe macrothrombocytopenia accompanied by platelet dysfunction, while in Gata1cKO^MK^ mice the red blood cells and white blood cell counts are unaffected. Our platelet functionality tests showed that our mouse model has similar platelet defects shown in previous Gata1 deficient mouse models, including a defect in aggregation when platelets are stimulated with botrocetin and collagen. This aggregation defect could be explained by the defective expression of respective receptors/subunits (*i*.*e*. vWF-R and CD49b). Besides these known platelet defects we show that platelets from Gata1cKO^MK^ mice have an aggregation defect when stimulated with aggretin and convulxin. Since the defect could not be explained by downregulation of Clec2 or GPVI receptor expression (or at least considering the extent of downregulation) on these platelets, we next aimed at studying whether a common signaling protein, Syk, could be involved in the lower response. In fact, FCA performed on SykcKO^MK^ platelets confirmed an essential role of Syk kinase on the platelet aggregation responses induced by aggretin, convulxin and collagen, but not on those induced by PMA or botrocetin. In addition, pre-treatment of platelets from WT animals with a Syk kinase inhibitor prior platelet aggregation induction results in the same FCA profile when using the same battery of agonists, *i*.*e*. defective aggretin, convulxin and collagen responses, normal PMA and botrocetin responses [29]. Syk, is essential during megakaryocyte migration and spreading and involved in platelet aggregation, and thus essential for normal megakaryocyte function [46]. We therefore found it of relevance to study whether Gata1 could be directly involved in the transcriptional regulation of Syk.

A reduced expression of Syk was noticed in mRNA of megakaryocytes and protein extracts of platelets from Gata1cKO^MK^ mice and we found that Gata1 directly regulates the transcription of Syk by employing ChIP assays. These data support the notion that Syk is a direct Gata1 target in megakaryocytes and explain the GPVI/Clec2 dependent aggregation defects shown in Gata1cKO^MK^ platelets. The lowered, but not complete absence of this protein could be explained by the fact that Gata1 recombination occurs in mature megakaryocytes, and we cannot exclude the possibility that combinatorial interactions between several transcription factors during megakaryopoiesis allows for basal transcription [12, 15, 18].

The severe thrombocytopenia caused a mild shift in megakaryocyte maturation in the bone marrow compartment, which could be explained by the increased demand in output of platelets. Interesting, however, is the new II+ population we identified in both the bone marrow and the splenic compartment. This SSC^low^ CD61^high^ population could be a consequence of the increased pressure on megakaryocyte maturation. Of note, although the ploidy profile of this II+ megakaryocyte population in Gata1cKO^MK^ mice resembles mostly the WT^lox^ II megakaryocyte ploidy profile, we have to consider that the 8N in the Gata1cKO^MK^ megakaryocytes displays a characteristic double peak, which does not appear in WT^lox^ samples.

Analysis of the hematopoietic precursor compartment revealed a shift in total number of MPs, CMPs, GMPs and MEPs. The shift could potentially be explained by the severe and chronic thrombocytopenia suffered by Gata1cKO^MK^ mice; still we could not discard Gata1-dependent or -independent alterations in relevant factors such as Pf4 or TPO to be causative of the misbalance observed at the progenitor level. Thrombocytopenia is normally accompanied by raised TPO levels (when the megakaryocyte-platelet mass is reduced), and it has been previously reported that besides a direct effect of TPO on megakaryocyte proliferation and maturation, TPO also influences directly hematopoietic stem cell (HSC) quiescence and cycling [40, 47]. Contrary to what could be anticipated, TPO levels were mildly lower in Gata1cKO^MK^ mice. This supports the notion that despite the severe thrombocytopenia, the megakaryocyte-platelet mass is increased due to the overgrowth of immature megakaryocytes. In concordance with these results, it has been previously reported that Gata1-low mice display normal TPO levels [48]. Reduction of MEPs could be a consequence of a higher demand of platelets, which leads to exhaustion of the most committed progenitors towards the demanded lineage, through TPO stimulation. In addition, we examined mRNA levels of Gata1-target Pf4 in bone marrow derived cultured megakaryocytes, as it has been recently reported the important and local role of this megakaryocyte-secreted factor on HSC senescence/performance [41]. Pf4 levels were increased in Gata1cKO^MK^ megakaryocytes. Interestingly, megakaryocytes from Gata1-low mice have increased Pf4 levels [49], profile that is reversed (Gata1 increases and Pf4 decreases) in Gata1-low mice treated with a TGFβ inhibitor. Furthermore, Gata1 timely represses Pf4 in immature megakaryocytes in cooperation with Eto2 [42]. It is important to acknowledge the complexity of Gata1 transcription regulation, which includes its participation in activating as well as repressing transcription factor complexes, as it has been well characterized in the erythroid lineage [13, 50].

A closer look at the erythroid compartment, revealed a change in erythroid maturation in the bone marrow and the spleen, and splenomegaly. Both organs showed an increased percentage of mature erythroid cells, which together with the splenomegaly, suggest compensatory stress erythropoiesis or extramedullary hematopoiesis/erythropoiesis that develops as a direct consequence of the (chronic) thrombocytopenia.

In summary, Gata1 loss in the megakaryocytic lineage leads to -chronic- thrombocytopenia and dysfunctional platelets. The platelet dysfunction is a direct consequence of Gata1 loss, since several receptors and signaling molecules are *bona fide* Gata1 targets, amongst which, Syk is demonstrated in the present manuscript. Megakaryopoiesis in these mice do not follow the normal paths, and Gata1 null megakaryocytes seem to proliferate rather than differentiate, as has been previously suggested. In the present manuscript we identify an aberrant megakaryocyte differentiation stage, misbalance of hematopoietic precursor progenitors and extramedullary hematopoiesis. We think that these latter events are a consequence of the -chronic- thrombocytopenia, rather than *Gata1* gene recombination in the megakaryocytic lineage itself. However, whether these events occur only in the context of Gata1 loss or are merely due to the severe prolonged thrombocytopenia (or both), needs to be further investigated. In particular, whether a local recruitment of TPO or overproduction of Pf4 by immature megakaryocytes would exert locally an effect on hematopoietic stem cells at the microenvironment level, remains to be elucidated.
